# Single-stage long-stem total knee arthroplasty in severe arthritis with stress fracture: a systematic review

**DOI:** 10.1186/s43019-023-00178-2

**Published:** 2023-01-19

**Authors:** Shubhankar Shekhar, Alok Rai, Saket Prakash, Tarun khare, Rajesh Malhotra

**Affiliations:** Department of Orthopaedics, All India Institute of Medical Sciences, New Delhi, India

## Abstract

**Purpose:**

Proximal tibia stress fractures present a challenge when performing total knee arthroplasty (TKA) in knee arthritis (KA). The literature on treatment modalities for stress fractures with arthritis is varied and not systematically reviewed. We aimed to answer the questions: (1) Is long-stem TKA sufficient for stress fractures in arthritic knees? (2) Should stress fracture and KA be addressed simultaneously? (3) What is the role of augmentative procedures in stress fractures with knee arthritis? (4) Can a unified algorithm be established?

**Methods:**

The PubMed and Cochrane databases were searched for keywords such as stress fracture, knee arthritis and total knee arthroplasty, published from January 1995 to 29 May 2022. A total of 472 records were screened down to 13 articles on the basis of our selection criteria. Ten data items were recorded from the included studies. The methodological index for non-randomised studies (MINORS) score for the included studies was 17 ± 3.

**Results:**

We found long-stem TKA to be sufficient for most cases and advocated for single-stage treatment of stress fractures and arthritis. Augmentative procedures play a role in the treatment, and a unified algorithm was drafted to guide treatment.

**Conclusion:**

Single-stage management of advanced KA with a stress fracture causes less morbidity than a staged procedure. Long-stem TKA, with or without an augmentative procedure, is an excellent option.

## Introduction

**Fig. 1:**
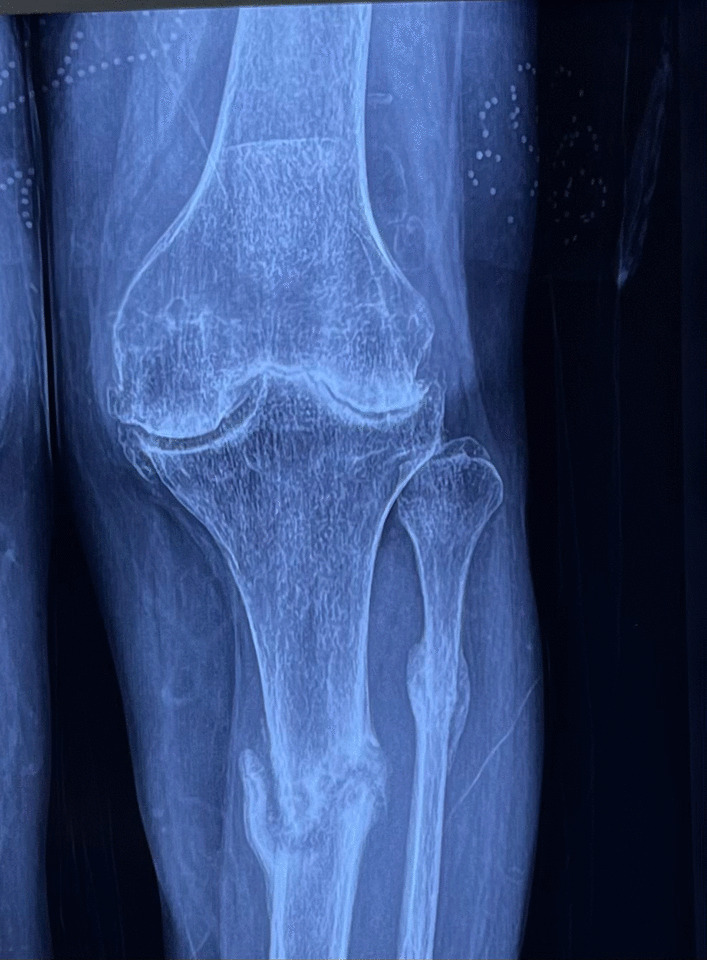
Antero-posterior (AP) and lateral X-ray showing severe knee arthritis with proximal tibia stress fracture (extra-articular)

**Fig. 2:**
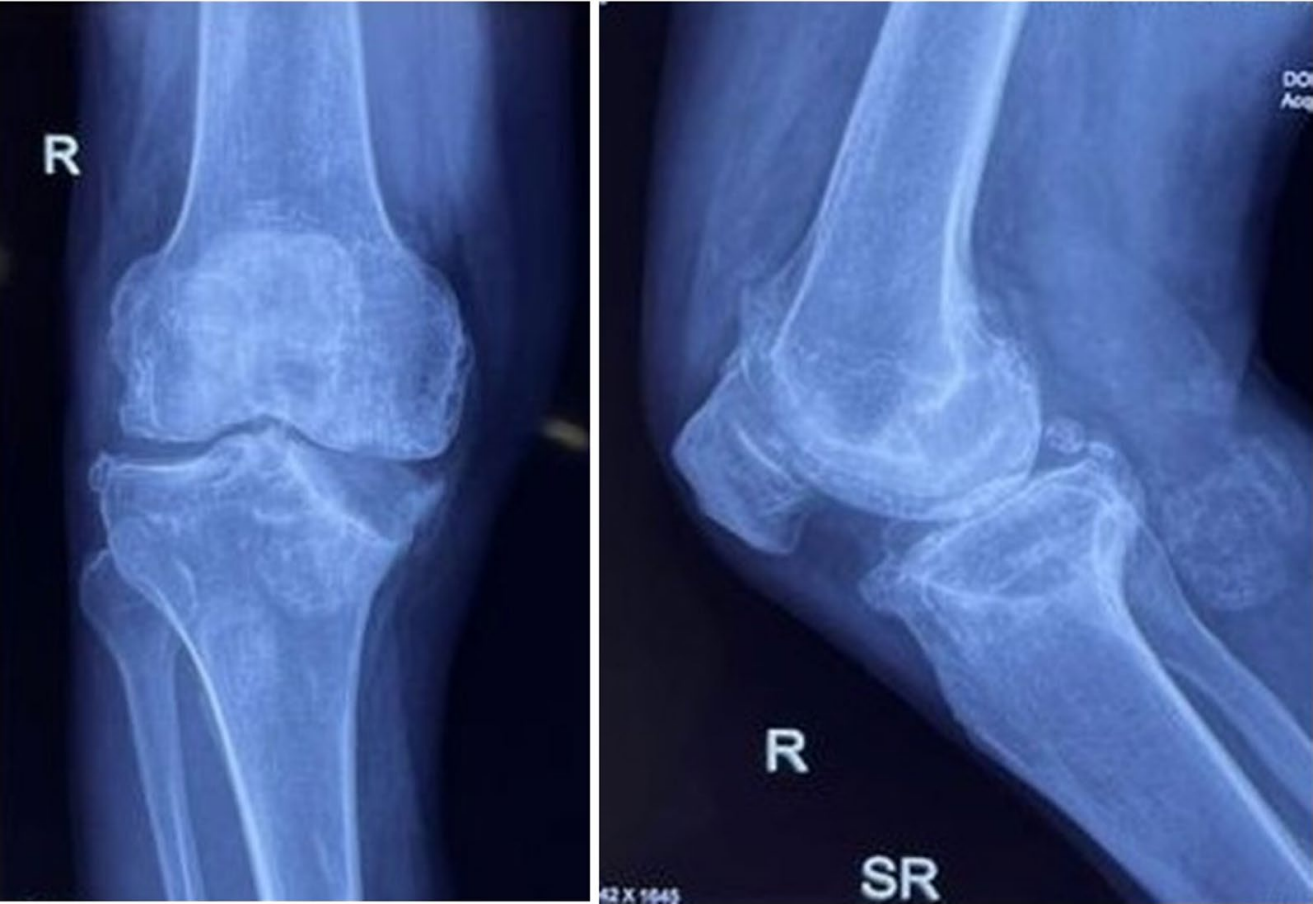
Antero-posterior (AP) and lateral X-ray showing severe knee arthritis with proximal tibia stress fracture (intra-articular)

Total knee arthroplasty (TKA) is the gold standard for end-stage knee arthritis (KA) [1]. Long-standing end-stage arthritis can be complicated by a stress fracture of the proximal tibia (Figs. 1, 2). Wheeldon was the first to report stress fractures in patients with KA [2]. A stress fracture occurs due to abnormal loading of a normal bone (fatigue), normal loading of an abnormal bone (insufficiency) or a mix of both [3]. Coronal plane deformities associated with arthritis cause eccentric loading and stress concentration, leading to a stress fracture [4, 5]. Persistent malalignment predisposes to malunion, or non-union, of a stress fracture. X-ray is the imaging modality of choice for the baseline diagnosis of stress fracture. Acute stress fracture is easy to be neglected. In patients with acute-on-chronic pain, or with shin pain, in the proximal tibia, suspicion should be aroused . A magnetic resonance imaging (MRI)/computed tomography (CT)/three-phase bone scan can confirm the diagnosis [6]. While acute stress fracture may present as a single line, chronic stress fractures show subperiosteal bone formation [7].

Stress fractures associated with KA are more common in developing countries. Living with long-standing coronal plane deformity is commonly attributed to factors including lack of knowledge, fear of surgery, economic constraints and negligence towards KA [8, 9]. This causes a delay in surgery, which results in a progressive coronal plane deformity. Obesity, poor bone health and metabolic bone diseases are precipitating factors [3]. Proximal tibia stress fracture associated with KA usually presents with severe acute-on-chronic pain, leading to the inability to bear weight. Stress fractures cause discontinuity and deformity in the proximal tibia, making total knee arthroplasty more challenging. This presents a dilemma to surgeons whether to address them simultaneously or sequentially.

Management of proximal tibia stress fracture associated with severe KA is debatable, ranging from conservative management [10] to staged surgery involving corrective osteotomy followed by TKA [11, 12], simultaneous long-stem TKA and internal fixation of stress fracture using plating [13], the use of long stem TKA alone [14] or the use of any of the above with augmentative procedures such as proximal fibular resection (PFR) [15] and/or plating and bone grafting [9].

There has been an attempt to classify these fractures according to location (intra-articular versus extra-articular), fracture mobility, amount of deformity, and duration [9, 14], but this has added further complexity to an already rare entity. There is a paucity of literature about the management of stress fracture associated with severe KA. Very few publications state guidelines for management or have reported outcome analysis and potential complications. To the best of our knowledge, we did not find any meta-analysis or systematic review about stress fractures in severe arthritis.

Purpose of study: our purpose was driven by the following four questions:

1. Is long-stem TKA sufficient for stress fractures in arthritic knees?
2. Should stress fracture and knee arthritis be addressed simultaneously?
3. What is the role of augmentative procedures in stress fractures with knee arthritis?
4. Can a unified algorithm be established to treat stress fractures with knee arthritis?

## Materials and methods

This systematic review was conducted as per the guidelines of the Preferred Reporting Items for Systematic Reviews and Meta-Analysis statement (PRISMA) [16].

### Data and literature sources

We performed a Boolean search on PubMed and Cochrane databases, comprising the following keywords: “stress fracture”, “Knee arthritis” and “Total knee arthroplasty”. The search was conducted for all studies indexed on the databases from January 1995 to 29 May 2022. January 1995 was selected as a start date to exclude outdated literature. A combination of Stress fracture “AND” Knee arthritis, Stress Fracture “AND” Total Knee Arthroplasty was run as the search parameters in PubMed and Cochrane.

### Study selection

#### Eligibility criteria

Studies were included or excluded on the basis of the following criteria:

#### Inclusion criteria

Studies that focused on proximal tibia stress fractures in patients with knee arthritis.

#### Exclusion criteria

1. Studies focusing on stress fractures of sites other than the proximal tibia.
2. Studies that included pathological fractures or traumatic fractures.
3. Case reports with fewer than three patients.
4. Studies that included stress fractures occurring after arthroplasty.
5. Articles not published in English.

### Data extraction

Original articles were then retrieved from our institutional repository (including institutional access to the relevant journals). The full text from each article was read, and respective data were organised and analysed independently by three reviewers, and tabled in an Excel sheet. Excel sheets from all three reviewers were then compiled into a single document.

**Fig. 3:**
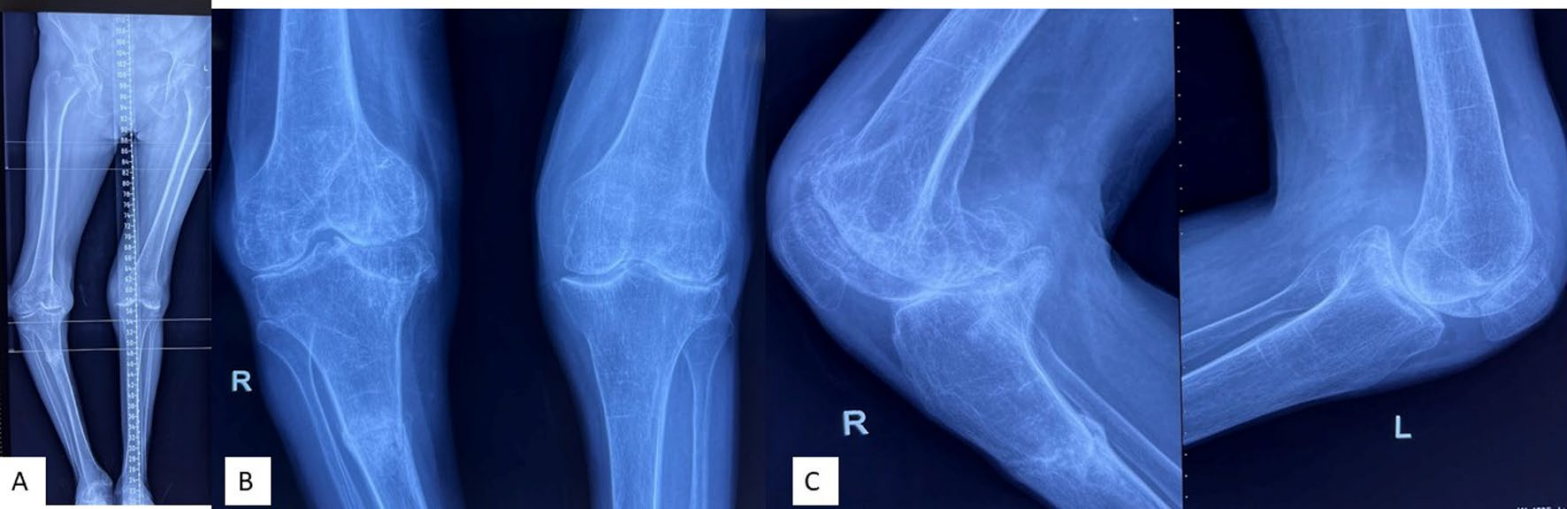
**A**–**C** Scanogram, AP and lateral view of osteoarthritis (OA) B/L knee with stress fracture (extra-articular) on right side

**Fig. 4:**
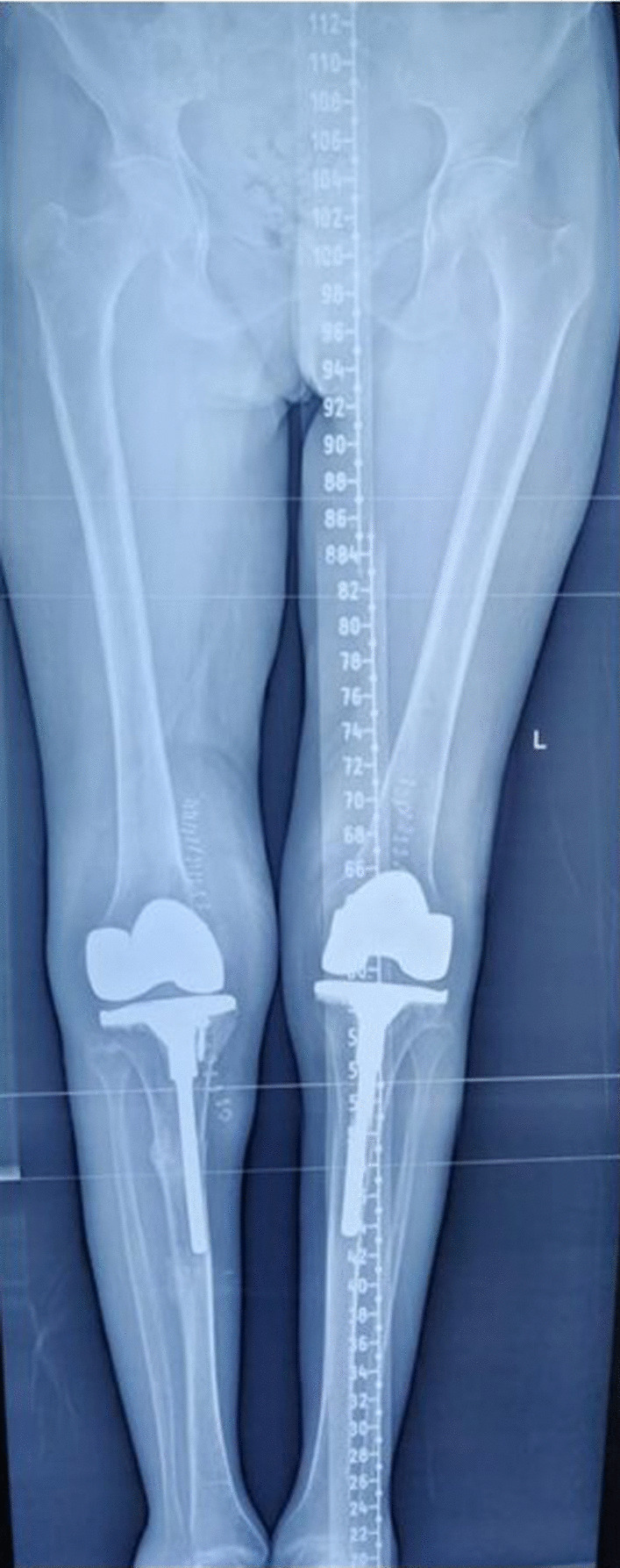
Post-operative scanogram

The following data were retrieved (where possible) from the selected studies:

1. The total number of patients.
2. Demographic parameters.
3. Aetiology of arthritis and number of patients in each study.
4. Body mass index (BMI) (available in 5 studies).
5. Pre-op knee deformity (available in 11 studies).
6. Post-op knee score (available in 11 studies).
7. Modality of treatment used (isolated TKA/long-stem TKA/corrective osteotomy + TKA/PFR + long-stem TKA/plating and bone grafting + long-stem TKA) (Figs. 3, 4).
8. Time to union.
9. Complications.
10. Type of implant used.

We also collected data on the authors, year and source of publication.

### Identification of studies

**Fig. 5:**
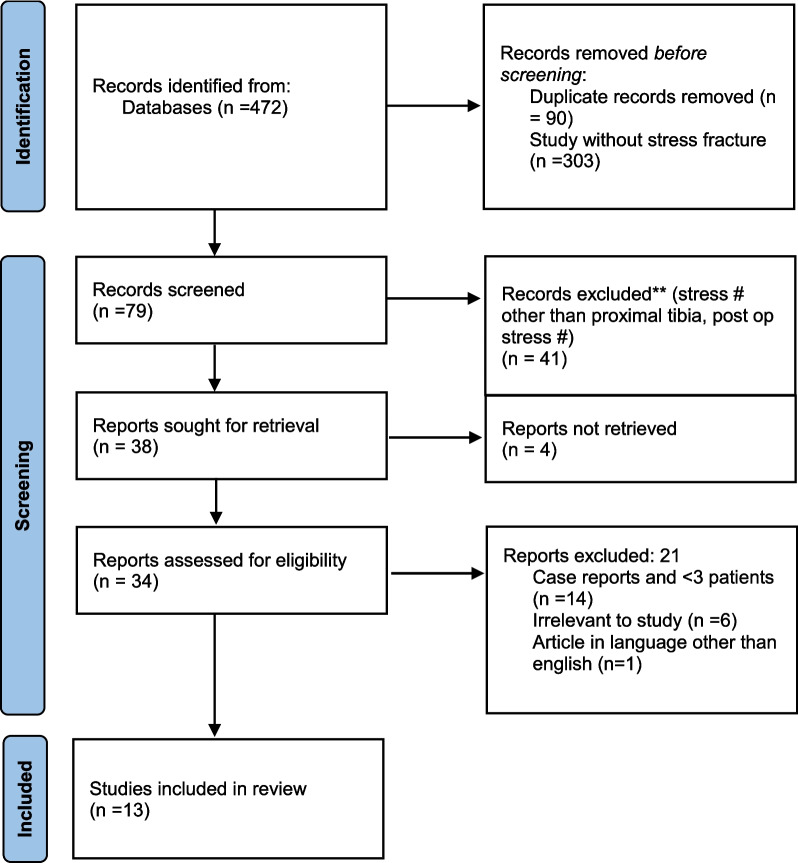
PRISMA flowchart showing the studies that were included in our review

Studies were identified from the search results. A total of 472 records were identified. Ninety duplicate records and 303 studies without stress fractures were excluded from screening, and three independent authors verified the exclusion. Seventy-nine articles were then screened by the same authors, independently. Forty-one records were excluded on the basis of this study’s criteria. Out of the remaining 38 articles, 4 could not be retrieved. Six articles were found to be irrelevant to the study. Case reports (*n* = 14) and articles in languages other than English (*n* = 1) were excluded. Thirteen studies were included in the review (Fig. 5).

### Assessment of methodological quality

MINORS was used to evaluate the quality of the included studies. Each item was scored as 0 (if not reported), 1 (when reported but inadequate) and 2 (when reported and adequate) [17].

The mean MINORS score was 17 ± 3 [mean standard deviation (SD) ± 2]. Major concerns were the lack of unbiased assessment of the study endpoint (no blinding), and only one randomised control study calculated sample size before the start of the study [15].

### Data synthesis and analysis

Risk assessment was done on the basis of demography. Cumulative data were analysed to determine treatment patterns and formulate a treatment algorithm. Results were reviewed independently to discuss implications. We looked for the reference range of different outcomes mentioned in the studies. Acceptable alignment post-TKA was considered according to the theory of mechanical alignment [18]. The Knee Society Score (KSS) is excellent if it lies between 80 and 100 [19, 20]. A meta-analysis was not performed because of the lack of homogeneous comparative studies. According to age groups and gender, analysis was not done because of the lack of normalised data across included studies. All data were collected, and outcomes were narrated.

## Results

**Fig. 6:**
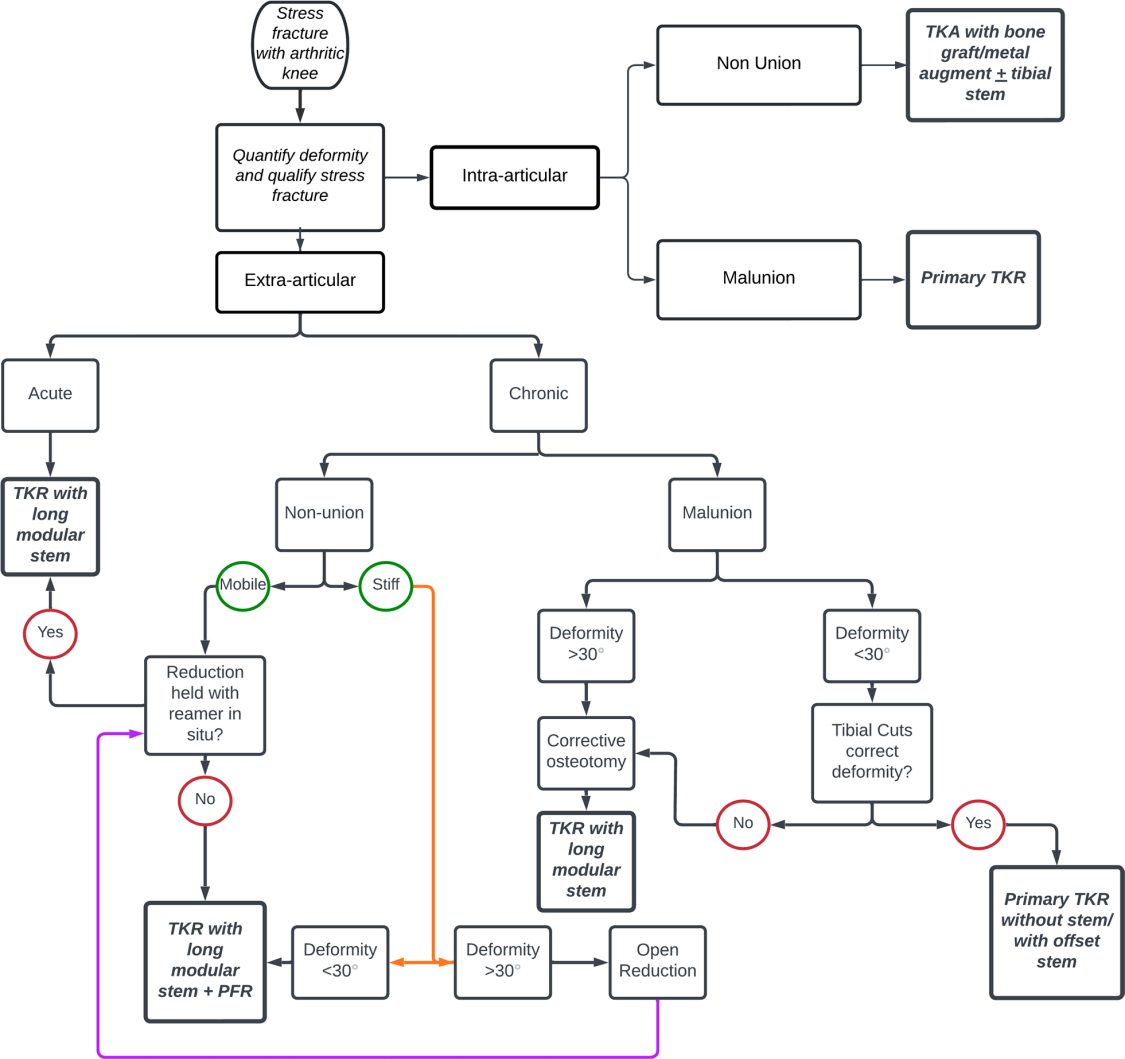
Showing unified treatment algorithm for management of stress fracture in proximal tibia after severe osteoarthritis

Our research questions and the attempts to answer them are as follows:

1. Is long-stem TKA sufficient for stress fractures in arthritic knees?
  Yes, in most cases, long-stem TKA is sufficient. Treatment is also dependent on the type of stress fracture, the degree of deformity and the tools available to a surgeon.
2. Should both stress fracture and knee arthritis be addressed simultaneously?
  Yes, as the treatment of both is interlinked, quite like their pathologies. Thus, a stress fracture and KA should be managed in a single-staged procedure.
3. What is the role of augmentative procedures in stress fractures with knee arthritis?
  Augmentative procedures such as PFR, osteotomy at the centre of rotation and angulation (CORA) of deformity, and plating and bone grafting play a role in malunion and severe deformity to (a) correct deformity and (b) maintain alignment.
4. Can a unified algorithm be established to treat stress fractures with knee arthritis?
  Yes, after going through available literature, we have drafted a unified treatment algorithm (Fig. 6). Though this may serve as a guiding light, the treatment decision should depend on the treating surgeon’s experience and expertise, and be patient specific.

Data on deformity correction, knee scores, implant used, procedures performed and complications have been summarised in Tables 1 and 2.

**Table 1:** Demographic details with deformity and knee scores

S. no	Study	Year	Number of patients (*n*) (M/F)	Number of knees	Mean (age)	Aetiology (RA/OA) (*n*)	Pre-op deformity		Post-op correction		Knee scores	
							Varus deformity (mean)	Valgus deformity (mean)	Varus	Valgus	Pre-op	Post-op
1	Tomlinson et al. [30]	1995	5 (0/5)	5	75	1/4	17.5	25				
2	Sawant et al. [31]	1999	4 (0/4)	4	72	1/3		16.75				
3	Moskal et al. [13]	2000	3 (0/3)	3	61.6	0/3	13				33^a^	84.3^a^
4	Mullaji et al. [14]	2008	34 (4/30)	42	63	5/37	20.8	24.6	2.2		36.7^a^	90.3^a^
5	Dhillon et al. [8]	2011	8 (3/5)	8	70.2	0/8					23.6^a^	80.8^a^
6	Mittal et al [21]	2013	29 (11/18)	31	66		23.2		1.9		38.5^a^	89.6^a^
7	Jabalameli et al. [20]	2017	17 (0/17)	17	68.1	3/14	20.9 ± 1.7					86.4 ± 2.8^a^
8	Soundarrajan et al. [26]	2018	20 (3/17)	20	64	0/20	18.2			1.8	21.9^a^	82.8^a^
9	Rashid et al. [28]	2018	15 (1/14)	15	65	3/12	20 ± 5	10 ± 2.5			31.5^a^	87.6^a^
10	Shah et al. [15]	2020	62 (20/42)	62	71.6	0/62	18.9 ± 1 (study), 18.3 ± 1.4 (control)		1.8 ± 3.1 (control)	1.7 (study)	54.7 ± 2.0^b^ (control), 54.1 ± 1.8^b^ (study)	26.9 ± 2.6^b^ (control), 19.9 ± 1.9^b^ (study)
11	Gill et al. [9]	2021	24 (0/24)	24	62.3	4/20	20.5 ± 3 (*n* = 21)	22.3 ± 4.6 (*n* = 3)	0.8 ± 0.4 (*n* = 21)	0.8 ± 0.28 (*n* = 3)	29.8 ± 6.1^a^ (group 1), 27.2 ± 3.3^a^ (group 2)	91.5 ± 4.8^a^ (group 1), 89.8 ± 3.8^a^ (group 2)
12	Pai et al. [25]	2022	12 (4/8)	12	67.3	0/12					32.9^a^	89.3^a^
13	Reddy et al. [27]	2022	17 (4/13)	17	65.3	0/17	21.2 ± 8.4		1.2 ± 1.6		18.9 ± 5.5^a^	89.4 ± 7.5^a^

^a^Knee score

^b^WOMAC score

**Table 2:** Treatment used in reviewed papers and complications

Study	Management							Type of implant used	Time of union	Complications
	TKA	Conservative	Long-stem TKA (LST)	LST + osteotomy	LST + plating	LST + PFO	LST + PFO + PLATING			
Tomlinson et al. [30]			5					PS		
Sawant et al. [31]			3	1				PS		
Moskal et al. [13]					1		2	PS		
Mullaji et al. [14]	18		13	5		5		PS		
Dhillon et al. [8]			6		2			PS	17.3–21.7 weeks	Superficial skin necrosis (*n* = 1) Stress concentration at stem tip (*n* = 1)
Mittal et al. [21]			28	3				PS	All united at last follow-up	
Jabalameli et al. [20]			17					LCCK 5 GENDER 7 LPS 5	8.3 ± 1.1 weeks	
Soundarrajan et al. [26]			18	2				PS PFC 19 GEN II 1	17.3–26 weeks	
Rashid et al. [28]			15					PS	19.5 ± 2.6 weeks	Pulmonary embolism (*n* = 1) Stroke (*n* = 1)
Shah et al. [15]			31			31	5*	PS PFC	12.2 ± 1.2 weeks 21.1 ± 5.16 weeks	
Gill et al. [9]			13				11	PS 16 CCK 5 RHK 3	20.4 weeks	Anterior tibial cortex perforation (*n* = 1)
Pai et al. [25]			12					CR/AS SIGMA 5 GEN II 7	9.4 weeks	
Reddy et al. [27]			17					PS PFC	10.2 ± 2.8 weeks	

*TKA* total knee arthroplasty, *LST* long-stem total knee arthroplasty, *PFO* proximal fibular osteotomy *PS* posterior stabilised, * 5 patient underwent second surgery needing plating

### Study characteristics

Most of the articles confirmed long-stem TKA as an adequate treatment. Two articles classified stress fractures and graded the need for proximal fibular resection with long-stem TKA [14, 21]. The authors advocated for osteotomy at the malunion site in case of extra-articular deformity > 30°, as it could not be addressed only with intra-articular resection [14, 21, 22]. Gill et al. classified patients similarly to Mullaji et al., but used plating and bone grafting in conjunction with TKA (with more reported complications in contrast to the other) [9, 14].Shah et al. conducted a prospective randomised controlled trial with a sample size of 62 [15]. In the control group (*n* = 31) (conventional treatment without fibular resection), the authors found delayed union and non-union in five patients and advocated for PFR for an early union. Pre-operative knee deformity was mentioned in 11 studies, but only 6 discussed post-operative deformity. In their article, Shah et al. mentioned post-operative deformity and reported 1.7° valgus in the study group (long-stem TKA with PFR), whereas the controls had 1.8 + 3.1° varus. Post-operative knee scores were available in 11 studies, all of which achieved excellent results (Table 1). BMI was taken into account in five studies, out of which it was > 30 kg/m^2^ in three studies.

KSS [23] (*n* = 10) and Western Ontario and McMaster Universities Arthritis index (WOMAC) [24] score (*n* = 1) were calculated in 11 studies. All of them reported good results with or without augmentative procedures.

### Aetiology

The included studies examined 250 patients and 260 knees with a mean age of 67.0 years. Eighty per cent of the patients (200 out of 250) were women (Table 1). Except for Mittal et al. [21], the articles mentioned aetiology, with OA being 92.5% (*n* = 212) and the rest rheumatoid arthritis (RA) (*n* = 17).

Implant design: all studies used a posterior stabilised (PS) implant design, except Pai et al. [25], who used a cruciate-retaining (CR) implant. Gill et al. (*n* = 5 minor) and Jabalameli et al. (*n* = 5) reported that the use of constrained implants was required [9, 20].

### Stem length

Every author has described that the stem should cross the fracture site, but no specific length description has been given. Pai et al. stated that the stem length should exceed the fracture site by a distance of at least two cortical diameters [25]. However, it is understandable that stem length can be more due to design differences among different manufacturers. Soundarrajan et al. stated that stem length should be planned preoperatively to cross the fracture site (due to said variations provided by different implant designs) [26].

### Cemented/uncemented stem

All studies used uncemented stems but cemented the tibial base plates. Cementing of the proximal part of the stem is debatable. Some advocate cementing it [14, 20, 21], while others cemented only the tibial base plate [25–27]. More importantly, the consensus was that care must be taken to prevent cement from entering the fracture site.

### Time to union of stress fracture

The radiological union was considered when bony continuity in three out of four cortices re-established. We did not find any reference range for the time to union of stress fractures of the proximal tibia. Therefore, we looked at the time to union in our included studies, which ranges between 7.2 and 26.1 weeks.

### Rehabilitation protocol

Patients were allowed full weight-bearing from post-operative day zero by Reddy et al. [27], Rashid et al. [28], Jablameli et al. [20] and Mittal et al. [21]. Partial weight-bearing was allowed by Pai et al. [25], Dhillon  et al. [8], Mullaji et al. [14] and Soundarrajan et al. [26] for 4–6 weeks. Mobile stress fractures (with only long-stem TKA as treatment) in the study by Gill et al. [9] were made to walk full weight-bearing with a knee immobiliser, while the patients in the other group (requiring long-stem TKA with plating) were made to walk non-weight-bearing for 4 weeks. Similarly, Soundarrajan et al. [26] kept patients who underwent plating non-weight-bearing for 4–6 weeks. All studies started knee range of motion and quadriceps strengthening exercises immediately following surgery.

## Discussion

Severe knee arthritis with obesity and concomitant osteoporosis are risk factors for stress fractures. Single-stage management of advanced KA with a stress fracture causes less morbidity than a staged procedure. Long-stem TKA, with or without an augmentative procedure, is an excellent option and is associated with good outcomes (early fracture union, stable correction of severe deformity, and early patient ambulation). The posterior cruciate ligament may be sacrificed to correct severe deformity; thus, a PS implant should be kept as a backup. Efforts should be made to treat cases as early as possible, as early detection and intervention are the keys to prevent disease progression and deformity. Care must be taken to ensure no distraction at the fracture site in order to prevent the occurrence of non-union. Thorough clinical evaluation, laboratory evaluation and careful preoperative planning are essential in managing this complex conundrum. A wholesome and inclusive approach should be used to address the interlinked pathologies, thus breaking a vicious cycle.

Stress fractures are reported in 1.3% of patients with KA and are more frequently seen in the South-East Asian subcontinent [8]. These stress fractures are usually the result of altered weight-bearing, coupled with poor bone quality [3]. Most patients do not seek treatment for disabilities owing to these fractures. While the pain caused may be attributed to a variety of non-scientific reasons, the fact remains that a lack of knowledge about the complications of the deformity caused by the fractures makes timely treatment improbable. Bone mineral density was measured only by Gill et al. [9], who found osteoporosis in 9 and osteopenia in 10 out of the 24 patients. Osteoporosis carries with it a significant economic burden [29]. Since a stress fracture can occur due to fatigue (insufficiency) of the proximal tibia, patients should be worked up for altered bone metabolism, and the treatment offered should be wholesome and inclusive .

Mullaji et al. classified stress fractures into intra-articular and extra-articular fractures [14]. The authors further subclassified intra-articular into malunited and united, and extra-articular into impending, acute, uniting, malunited and united. A closed-wedge osteotomy and TKA with tibial stem extender in malunited extra-articular deformity, and debridement in conjunction with TKA with tibial stem extender and  segmental fibulectomy, were performed in cases with non-union [14]. The opinions of authors included in the study were used to tackle the lack of consensus on treatment of stress fractures with KA. Fracture stability is an important concern and has been tackled in multiple ways. Fluoroscopy was used intraoperatively to check alignment, guide and check reaming in severe deformity and ascertain stem length [8, 14, 25, 26].

Shah et al. noted delayed union and non-union in five patients in the control group and advocated for PFR for early union [15]. However, in contrast, three studies have reported an earlier union without PFR [20, 25, 27]. Though the role of PFR remains inconclusive for early union, it may be helpful in stiff non-unions for the correction of the deformity.

A wholesome approach to treating stress fractures in arthritic knees in a single sitting has been advocated by all modern studies. TKA in conjunction with modular stems, with or without augmentative procedures, shows a good outcome. Early mobilisation, coupled with excellent KSS, makes outcomes of TKA in patients with stress fractures comparable to those of TKA performed in patients without stress fractures [32].

## Limitations

The quality of this systematic review is inherently related to the quality of the included studies. While there was only one level I comparative clinical trial, the rest were level II, III and IV studies. This heterogeneity and lack of level I studies were significant shortcomings of our systematic review. Patient selection bias/uncontrolled confounding factors are more common in level III and IV studies. There were not enough homogeneous comparative studies available, which precluded us from analysing the results with a meta-analysis. Our analysis also could not control several factors that might have influenced the outcomes, such as various patient characteristics and implant designs used for treatment. To avoid the influence of this heterogeneity on our results, we decided not to pool the results and only report the values as a range. Limited studies have stated the treatment concerning the severity of the deformity. In the spirit of evidence-based medicine, these drawbacks should be analysed and reported in future studies to help guide an orthopaedic surgeon and better address patients’ expectations.

## Conclusion

Single-stage management of advanced KA with stress fracture causes less morbidity than a staged procedure. Long-stem TKA, with or without an augmentative procedure, is an excellent option.

## Data Availability

All data generated or analysed during this study are included in this published article.
